# Clinical learning opportunity in public academic hospitals: A concept analysis

**DOI:** 10.4102/hsag.v27i0.1920

**Published:** 2022-10-26

**Authors:** Mpho N. Motsaanaka, Agnes Makhene, Gugu Ndawo

**Affiliations:** 1 Department of Nursing, Faculty of Health Sciences, University of Johannesburg, Johannesburg, South Africa

**Keywords:** concept analysis, clinical learning opportunity, defining attributes, antecedents, consequences, student nurses

## Abstract

**Background:**

Clinical learning opportunities (CLO) are vital educational encounters occurring in various clinical areas to provide the student nurses with clinical knowledge and experiences to develop their competencies for professional practice. However, CLO is a broad concept with varied characteristics that allow ambiguity, limiting its understanding and use. Its ambiguous nature leads to uncertainties and poor development of the required clinical attributes of successful theory to practice integration, higher-order thinking skills (HOTS) and clinical competencies.

**Aim:**

The aim of the study was to explore and describe the conceptual meaning of CLO, have a clear understanding and insight into the concept and identify the antecedents and consequences for pragmatic purposes.

**Setting:**

The study took place in a public academic hospital in Gauteng.

**Methods:**

The eight steps of concept analysis by Walker and Avant were followed. Multiple data sources not limited to nursing were explored and critically analysed for the definitions, characteristics, nature and uses of CLO from different fields of study.

**Results:**

The defining attributes of CLO were identified, namely the context, antecedents, processes, consequences and outcomes. The context within which CLO occurs was dynamic, multidimensional, real-life healthcare settings; antecedents included planning of clinical placement, provision of learning outcomes and consideration of the cognitive level of the students. Engagement and active participation in collaborative, problem-based learning activities, community-based research and the use of emerging technologies were the processes identified. The outcomes were autonomous, confident, competent professional nurses with critical thinking, clinical reasoning, judgement, critical decision making and problem-solving skills.

**Conclusion:**

A theoretical and operational definition of CLO was developed. The findings and results of concept analysis identified and specified the defining attributes of clinical learning opportunity. The findings can assist nurse educators, Clinical Education and Teaching Unit (CETU) personnel and operational managers to enhance CLO for student nurses to achieve their clinical learning goals and outcomes. The evaluation tools that may be adopted to assess the acquired clinical skills were also identified.

**Contribution:**

An increase in the existing body of knowledge in nursing education, considering that enhancing the CLO exposes students to various clinical experiences contributing to their development of clinical competencies to solve complex problems. The strategies to enhance the CLO will be developed, which may also provide vital information for policy development. Conceptualisation of the findings to nursing practice and quality patient care will be integrated into relevant literature.

## Introduction

Nursing is a practice-based profession that requires adequate clinical learning and positive practical experiences to prepare the student nurses for their professional roles. Musafiri and Daniels (2020) report that clinical learning opportunities (CLOs) are approaches for student nurses engaged in real-life clinical activities for meaningful experiences. Salifu, Heymans and Christmals (2022) added that the clinical placement of student nurses is vital because it provides them with adequate exposure and CLOs in real-life practice settings, as well as motivation to achieve their clinical learning outcomes. However, there is overcrowding of students in the public academic hospital under study, caused by the placement of students from different nursing education institutions as well as students from other health disciplines (Motsaanaka, Makhene & Ally 2020). The overcrowding leads to inadequate CLOs for the student nurses, with the resultant inability to integrate theory to practice, poor development of critical skills and clinical competencies, and a lack of professional growth (Mbakaya et al. 2020). Furthermore, student nurses became despondent and demotivated, leading to frustration, increased absenteeism and prolonged length of study due to inadequate CLOs (Berhe & Gebretensaye 2021; Motsaanaka et al. 2020). Exposure to real-life practice settings, experiences and clinical activities provides opportunities that will facilitate clinical learning to bridge the gap between theory and practice and prepare the student nurses to develop the qualifications of a clinically competent, independent, professional nurse practitioner (Taylor et al. 2021; Zulu, Du Plessis & Koen 2021). Motsaanaka et al. (2020) also report that clinical exposure to learning opportunities will develop the student nurses’ clinical competencies in higher-order thinking skills (HOTS). They will also help them with clinical preparedness as they undergo role transition into the nursing profession. It is also a learning period where student nurses demonstrate applied competency in integrating foundational, practical and reflexive competencies in real-life healthcare settings, as stipulated by the *South African Qualifications Act* (No. 58 of 1995) (Republic of South Africa [RSA] 1995). Mothiba, Bopape and Mbombi (2020) suggest that nursing education institutions need to design and implement clinical learning activities that will provide the student nurses with opportunities to develop the required professional skills and clinical competence. The student nurses will have confidence in their ability to provide and achieve quality health care for the patients (Al-Harahsheh et al. 2020) Implementing CLOs and activities will also improve the quality of clinical education for the student nurses and prepare them to practise in the 21st century (Modarres et al. 2021).

Despite the critical discussions on the importance and various applications of CLOs in literature, there is a lack of clarity in its definition and understanding as a concept (Stoffels et al. 2021; Walker & Avant 2019). A lack of clear definition and misunderstanding of the concept created challenges for nursing educators, clinical education and training unit (CETU) personnel, and operational managers in providing student nurses with adequate exposure to clinical teaching, learning and positive practical experiences (Taylor et al. 2021). The ambiguous and abstract nature of the concept of CLOs was defined and clarified to capture its contextual intricacies and to help improve the global standards of professional clinical education in the practice-based profession (Baker, Cary & Bento 2021; Shaterjalali et al. 2018; WHO 2020).

## Research method and design

The concept of clinical learning opportunity was analysed using the eight steps of concept analysis by Walker and Avant (2019). Extensive literature and multiple data sources, not limited to nursing, were consulted to explore and critically analyse the definitions, characteristics, nature and uses of clinical learning opportunities from different fields of study. Online dictionaries, encyclopaedias and thesauruses, as well as databases of Cumulative Index of Nursing and Allied Health Literature (CINAHL), Elton B. Stephens Company (EBSCOhost), PubMed Central (PMC), ScienceDirect and other online sources were searched to analyse and capture the meanings of the concept. The Google Scholar search engine was also consulted for relevant and related articles on the concept. Data saturation was reached after a total sample of 132 definitions, characteristics, nature and uses for critical analysis. Walker and Avant’s eight steps of concept analysis followed were (1) selecting a concept, (2) determining the aims of concept analysis, (3) identifying all uses of the concept, (4) determining the defining attributes, (5) identifying antecedents and consequences, (6) defining empirical referents, (7) identifying a model case and (8) additional cases.

### Step 1: Select a concept

When selecting the concept, the researcher began by identifying the nature of the problem to be addressed by the study (Rodgers, Jacelon & Kanafl 2018). The problem that drove the research study was the ambiguous and unclear meaning of clinical learning opportunity that created challenges and uncertainties for nurse educators, CETU personnel and operational managers in the clinical areas. The student nurses were not provided with sufficient CLOs and practical experiences to meet their clinical learning goals (Mothiba et al. 2020; Motsaanaka et al. 2020; Taylor et al. 2021). The uncertainties and challenges led to the poor professional development of the student nurses (Berhe & Gebretensaye 2021). The inadequate operational understanding and uncertainties of clinical learning opportunity directed the focus of the study for concept analysis and to address the research problem.

### Step 2: Determine the aims of concept analysis

Concept analysis aimed to clarify the conceptual meaning of clinical learning opportunities within the context of the study, have a clear understanding and insight into the concept and identify the antecedents and consequences for pragmatic purposes in the clinical settings (Walker & Avant 2019).

### Step 3: Identify the uses of the concept (sample and sampling method)

Extensive literature and multiple data sources (not limited to nursing) were consulted to explore and critically analyse the definitions, characteristics, nature and uses of clinical learning opportunities from different fields of study. The aim was not to limit the literature search but to avoid a biased understanding of the true nature and meaning of the concept (Walker & Avant 2019). The keywords used to explore the definitions of the concept were derived from the selected concept of ‘clinical learning opportunity’, which were ‘clinical’, ‘learning’ and ‘opportunity’. Online dictionaries, encyclopaedias and thesauruses were explored, as these sources do not describe clinical learning opportunity as a whole concept, and 15 definitions of the keywords were identified. The researcher continued to explore the databases of CINAHL, EBSCOhost, PMC, ScienceDirect, and other online sources using the keyword ‘clinical learning opportunity’ for critical analysis and capturing the meaning of the selected concept. A total number of 363 000 articles were found eligible for the definitions, characteristics, nature and uses of clinical learning opportunity. The resulting literature was initially searched and screened by reviewing titles and abstracts for relevance. The aim was to capture all the related meanings of the concept and increase the validity of the results. Literature searched was not limited to the years of publication.

From the 363 000 articles retrieved, 345 200 were not full-text articles; thus these were excluded, and 17 800 full-text articles remained for inclusion. Further, 16 188 articles were excluded as they had data already reported in other included articles, and 1612 full-text articles remained for inclusion. The redundancy of exploring the data sources occurred when the researcher had read 117 articles and reached data saturation. These articles were managed and authenticated via the Mendeley reference manager system. Data saturation is when other sources provided no added information, and a sense of closure is imminent (Polit & Beck 2019). The 117 articles were added to the 15 definitions of the concept from online dictionaries, encyclopaedias and thesauruses, resulting in the final list of 132 definitions, characteristics, nature and uses of clinical learning opportunities for review and critical analysis.

**FIGURE 1:**
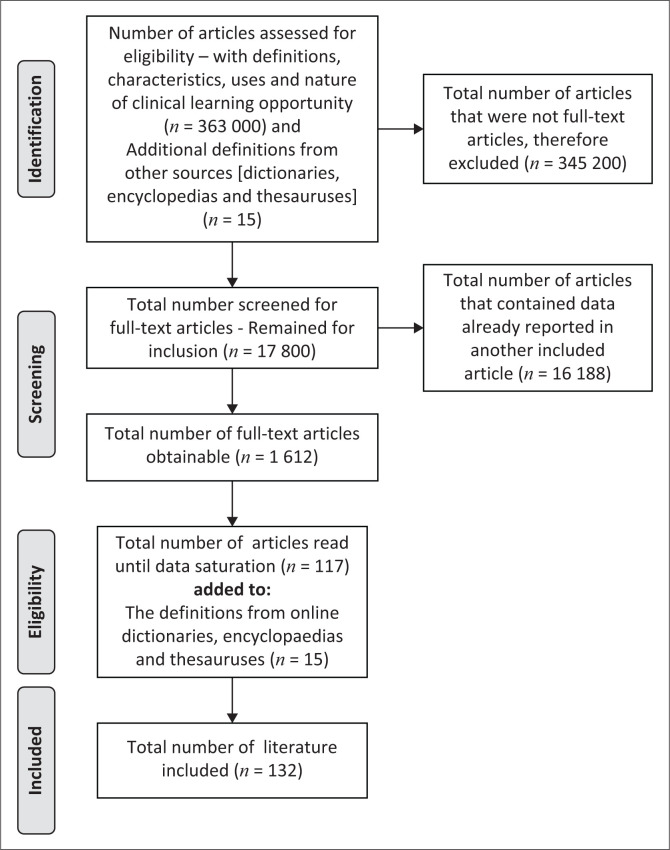
Preferred Reporting Items for Systematic Reviews and Meta-Analyses (PRISMA) flow diagram.

Figure 1 is a Preferred Reporting Items for Systematic Reviews and Meta-Analyses (PRISMA) flow diagram adopted to show the steps of identification, screening, eligibility, inclusions and exclusions of the literature in the study. The aim was to map the existing literature in the field of interest in terms of the volume, nature and characteristics of the primary research. It also ensured rigorous, transparent and complete reporting of the literature reviewed (Zhang et al. 2019). The flow diagram summarises all the empirical evidence that addressed the processes embarked on for the search of definitions, characteristics, nature and uses of the concept, thus minimising biases.

### Step 4: Determine the defining attributes (data collection method)

Determining the defining attributes of a concept is regarded as fundamental to describing its characteristics, as they contribute to a clear and in-depth understanding of the concept (Walker & Avant 2019). A three-columned table was used to identify the defining attributes. The first column displayed all the definitions, characteristics, nature and uses of the concept from various data sources. The researcher then manually and repetitively browsed through each literature to identify and underline similar and contrary patterns and phrases that appeared repeatedly. Similar and contrary patterns related closely to each other were clustered and classified together to formulate new and meaningful statements of clinical learning opportunities (content analysis). The process of content analysis assisted in the compilation and creation of 30 meaningful statements from the available literature to provide new insight and understanding of the concept (Roller 2019; Walker & Avant 2019). The formulated statements were transposed, redefined and categorised into the defining attributes of the concept, namely the context, antecedents, process, consequences and outcomes of CLO.

### Step 5: Identify antecedents and consequences (data analysis)

Antecedents are events that precede the occurrence of the concept (Walker & Avant 2019). Not revealing the antecedents could prevent the activity of clinical learning opportunity from occurring. The antecedents that must be in place were identified as planning of clinical placement, provision of clinical learning outcomes and consideration of the cognitive level of students. Consequences are the results of the occurrence of the concept (Walker & Avant 2019). The consequences of clinical learning opportunity were the development of HOTS and clinical competency, integration of theory and practice, provision of holistic patient care and the transition from novice to expert healthcare practitioners. The description of the defining attributes is presented under the findings and discussion to provide the importance of each.

### Step 6: Define the empirical referents (data analysis)

Empirical referents are classifications or categories; their presence determines the existence of the concept (Walker & Avant 2019). The empirical referents of clinical learning opportunity were recognised as higher-order skills such as critical thinking, clinical reasoning, judgement, critical decision-making and problem-solving skills.

Various authentic assessment tools are available to measure and evaluate the empirical referents and to verify the occurrence of clinical learning opportunity for the student nurses. The most common assessment tools explored, described and applied for all the identified empirical referents and within the context of the entire study are listed in Table 1. The assessment tools can be adopted to evaluate the occurrence of the empirical referents of clinical learning opportunities for student nurses.

**TABLE 1:** Empirical referents of clinical learning opportunity and their assessment.

Empirical referents		Assessment tools
1.1	Critical thinking skills	California Critical Thinking Skills Test (CCTST) (Facione & Facione 1992) California Thinking Disposition Inventory (CTDI) (Facione & Facione 1992) Watson–Glaser Critical Thinking Appraisal (WGCTA) (Watson & Glaser 1920s) The Critical Thinking Scale (CTS) (Ennis & Weir 1985)
1.2	Clinical reasoning skills	Nurses Clinical Reasoning Scale (NCRS) (Martinez De Castillo 2014) Clinical Reasoning Model (CRM) (Levett-Jones et al. 2010) Script Concordance Testing (SCT) and Extended matching Questions (EMQs) (Gagnon et al. 2009)
1.3	Clinical judgement skills	Clinical Judgment Model (CJM) (Tanner 2006) Lasater Clinical Judgment Rubric (LCJR) (Lasater 2007)
1.4	Critical decision-making skills	Clinical Decision-making scale (CDMS) (Lauri & Salantera 2002) Matrix Decision-making Model and ‘PERSON’ evaluation tool (Standing 2020)
1.5	Problem-solving skills	Problem Solving Inventory (PSI) (Heppner & Peterson 1982) Problem Solving Value Rubric (PSVR) (Schmidt-McCormack 2017)

### Step 7: Identify a model case

The researcher formulated a practical model case (Box 1) to demonstrate the use of all the identified defining attributes of clinical learning opportunity within nursing education and clinical learning for better understanding (Walker & Avant 2019).

BOX 1 A model case.

Students from nursing, physiotherapy, pharmacy and medicine, as well as different educators and clinical experts from two countries, took part in interprofessional learning (IPL) and collaboration. The learning activities occurred in dynamic, multidimensional, real-life healthcare settings *(context)*. Relevant stakeholders engaged in discussions and decision-making on the detailed plan of IPL *(planning of clinical placement)*. They held multiple virtual sessions to identify and clarify inter-related professional roles and discussed common clinical learning goals *(provision of clinical learning outcomes)*. In collaboration, they formulated two groups of second-year student nurses, second-year physiotherapy, final-year pharmacy and fourth-year medical students *(students’ cognitive level)*.

All students engaged and actively participated in various learning activities, which involved interactive, cooperative and collective efforts *(c ollaborative activities)*. They worked together on complex real-life health problems in the clinical area, searching for solutions to address the problem *(problem-based learning [PBL])*. Students also engaged with the community in scientific investigations to address their health and social issues *(community-based research)*. They used smartphones, electronic health records, health information networks and patients’ portals to impact learning and healthcare in the 21st century *(emerging innovative technology)*.

The students learned to apply the acquired theoretical knowledge with rationales to guide their actions in various real-life clinical situations *(integration of theory and practice)*. They displayed different levels of insight through creative, critical, reflective and innovative thinking capabilities to make decisions and solve complex health problems (*HOTS*). Students demonstrated the integration of foundational, practical and reflexive skills *(clinical competency)*. They delivered comprehensive care to the patients based on a mutual understanding of their physical, psychological, emotional and spiritual dimensions *(provision of holistic patient care)*. The students built a substantial scientific knowledge base, clinical expertise and experience to excel in their professional domains *(transition from novice to expert)*.

Students engaged in argumentation, dialogue and debate to analyse, make inferences and evaluate the data to reach in-depth understanding and insight *(critical thinking skills)*. They applied thinking strategies, knowledge and scientific evidence to deliberate, argue and justify their views and practices *(clinical reasoning skills)*. Students used their thought processes and clinical reasoning skills to prioritise decisions based on subjective and objective data *(clinical judgement)*. They integrated the acquired knowledge with scientifically evidence-based practices to decide on relevant interventions to solve patients’ complex health problems *(critical decision-making)*. They performed analysis to identify and define the patients’ health problems, implement practical solutions and evaluate outcomes *(problem-solving skills)*.

They developed the authority to assess and provide quality patient care based on competence, professional expertise and knowledge consistent with professional standards *(autonomous)*. They can independently care for and collaborate with other healthcare team members to effectively prioritise and coordinate patients’ daily care activities *(confident)*. Students practised ethically and fulfilled their professional responsibility by integrating the knowledge, skills, values, beliefs and acquired experience to provide effective patient care to cope with various clinical situations *(competent)*.

### Step 8: Identify additional cases

As a modification to Walker and Avant’s (2019) process of concept analysis, the additional cases of the borderline, related, invented and illegitimate cases were not constructed as the model case provided a clear understanding of the concept (Bourne, Smeltzer & Kelly 2021). In this study, only a contrary case was formulated to fully articulate, clarify and make a better judgement about the concept within the context of this study. A contrary case is the direct opposite of a model case, which indicated a clear example of what the concept was not and thus lacked all the defining attributes (Walker & Avant 2019).

#### A contrary case

The clinical learning opportunity is not experimentation, laboratory, or theoretical studies and does not include assigning the students to patient care unaccompanied.

### Ethical considerations

As stipulated in the *National Health Act* (No. 61 of 2003) (Republic of South Africa, 2003), ethical approval was obtained from the Research Ethics Committee (REC) and Higher Degree Committee (HDC) of the University of Johannesburg (reference number: REC-01-149-2019). Permission from the National Health Research Database (NHRD) and the Department of Health and the Research Approval Committee (RAC) to conduct the study in the public academic hospital in Gauteng was also granted. This article is a concept analysis.

## Discussion

The defining attributes of clinical learning opportunity discovered during the concept analysis were categorised as the context, antecedents, process, consequences and outcomes. Each defining attribute was explained, followed by the conceptual map and the theoretical definition of clinical learning opportunity.

### The context: Dynamic, multidimensional, real-life healthcare settings

Student nurses need clinical experiences from various healthcare settings that offer opportunities for successful learning. Such a learning environment needs to be stimulating, self-motivating and supportive with a diverse range of healthcare professionals, real-life patients and a complex psychosocial setting rich in learning resources (Van Rossem et al. 2019). A context with an interactive network of forces, complexity and exposure increases their clinical opportunities for interprofessional learning (IPL) enhances challenging experiences and helps students to develop various higher-order skills (Van Diggele et al. 2020). Moreover, it develops clinical competencies and the best scientific practices to solve complex problems within varied clinical healthcare settings. According to Nair, Thomas and Prem (2020), learning in a dynamic environment with a complex real-life health problem can be adapted with creativity and innovation, providing and promoting learning opportunities for interprofessional interactions, with the development of critical thinking, problem-solving and communication skills.

### The antecedent

#### Planning of clinical placement

Planning clinical placement is a strategic arrangement where student nurses would be allocated in a conducive and dynamic clinical setting practice environment that supports and enhances a positive interaction with learning experiences. Bøe and Debesay (2021) support the planning of clinical placement of student nurses to provide a supportive environment that ensures authentic experiences, adequate exposure and practice opportunities to achieve varying levels of proficiencies and the ability to respond to complex health problems. The appropriate clinical placement of student nurses fostered interprofessional collaborative learning, exposing them to diverse learning opportunities within the clinical practice environment (Aggar et al. 2020). Furthermore, it assisted student nurses to develop the knowledge, clinical skills and attributes to prepare them to become safe, competent and independent healthcare professionals (Atakro et al. 2020).

#### Provision of clinical learning outcomes

Providing clinical learning outcomes highlights clear and shared expectations between student nurses and clinical facilitators on professional abilities, skills and values that need to be achieved at the end of the student’s clinical learning experiences and practice (Hustad et al. 2019; Masilaca, Kumar & Balekiwai 2018). Clinical learning outcomes aligned with the qualification level registered on the National Qualifications Framework (NQF) ensured communication of course expectations to the student nurses in order to supply a seamless success and progression (SANC 2013). Providing clinical learning outcomes is beneficial to motivating students’ clinical learning associated with practice opportunities for a transparent, focused and successful pathway to achieving their goals.

#### Consideration of cognitive level of student nurses

Consideration of the cognitive level of student nurses is vital as it promotes building upon and combining their prior knowledge with the latest information to process new understanding that will develop their critical thinking, analysis and evaluation (Mi & Ok-Hee 2021; Nascimento et al. 2021). Taking into consideration their cognitive level can be used to support and apply constructive learning to know how best to assist and guide student nurses to develop intellectually and build their skills needed for effective learning. The more developed students are at higher-order skills, the better they are at learning and constructing new knowledge to apply in clinical practice. Upahi and Ramnarain (2020) highlighted the changing professional standards and health complexities that require nurse educators to create and provide student nurses with learning opportunities to develop higher-order cognitive skills through problem solving activities. Creative strategies and learning opportunities need to be tailored to students’ needs and progressively build their knowledge to perform the tasks in practice and further facilitate their progression from dependence to independence (Den Hertog & Boshuizen 2022).

### The process

#### Engagement and active participation in collaborative activities

Collaborative learning is an innovative, instructional small group learning approach in which students are required or encouraged to work together to enhance learning and achieve common learning goals (Markowski et al. 2021). Encouraging such activities will further allow shared information from different professional backgrounds, creating professional role clarity and an opportunity to cooperatively participate in constructing patient care plans to address their complex health needs (Chetty, Bangalee & Brysiewicz 2020; WHO 2020). Furthermore, students’ engagement and active participation in collaborative activities create an authentic learning experience that develops their understanding as they relate, synthesise, discuss, communicate and justify their ideas and thoughts within an IPL context (Chabeli, Nolte & Ndawo 2021). Interprofessional collaborative activities can enhance the students’ learning opportunities. They will acquire the necessary knowledge and transferable skills to become global health professionals with critical thinking, decision-making and problem-solving skills (Joseph 2018; Kim et al. 2019).

#### Engagement and active participation in problem-based learning activities

Engagement and active participation of student nurses in problem-based learning activities stimulate their critical thinking skills and enhance their ability to apply theory to practice (Wosinskic et al. 2018). The student nurses will develop problem-solving, leadership and teamwork skills. Seibert (2021) added that students learn to construct their knowledge, become creative and analytical, applying reasoning and problem-solving skills with opportunities to implement practical solutions to improve patients’ health and well-being. Problem-based learning activities aim to address complex health problems and challenges experienced and encountered in real-life situations, with a shift from a traditional teaching paradigm of teacher-directed and traditional lecture format to a learning paradigm of self-directed, interactive learning (Jensen, Srentoft & Ravn 2019).

#### Engagement and active participation in community-based research

Ghasemi, Moonaghi, and Heydari (2020) and Jacobs (2020) support the engagement and active participation of student nurses in community-based research as it promotes the collaborative development of community health projects, solving various and complex health conditions and morbidities. Community-based research removes the various social, cultural and logistical barriers that confound the well-intentioned efforts of public healthcare services (Chabeli et al. 2021; Tremblay et al. 2018). The intention is to provide learning opportunities for student nurses to develop a deeper understanding of global health issues and promote access to primary care, disease prevention and health-promoting behaviours, sustainable scientific evidence-based health practices and professional maturity (Vera 2020).

#### Engagement and active participation in emerging innovative technologies

Technology has transformed learning for student nurses into a more blended, interactive, and enhanced collaborative learning opportunities (Howlett & Waemusa 2019; Wittmann-Price, Wilson & Gittings 2020). The understanding and use of emerging technology will allow them to serve on the front lines of applying the latest advancements in scientific evidence-based research, allowing communication with their colleagues in real time and helping their patients with increased efficiency and effectiveness (Broughel & Thierer 2019; Carroll 2020). The educational goal of using technology facilitates and promotes CLO, improving clinical and psychomotor skills, especially on critical and rare skills.

## Consequences

### Higher-order thinking skills and clinical competency

Developing and stimulating HOTS and clinical competency in student nurses is crucial to everyday practice, as they equip them with the required skills to respond to and solve varied and complex real-life health problems within dynamic healthcare settings (Boso, Van Der Merwe & Gross 2021; Fukada 2018). Factors such as effective clinical learning environment, clinical experience, learning opportunities, motivation and theoretical knowledge are among the most important factors affecting clinical competency of nurses (Karami, Farokhzadian & Foroughameri 2017). Student nurses with HOTS can make clinical judgements and critical decisions in providing quality patient care in a complex, dynamic healthcare environment with various challenges and demands.

### Integration of theory and practice

Student nurses need to learn and apply the theoretical framework into practice effectively and confidently to become competent healthcare professionals. Integrating theory and practice is vital as it shows student nurses’ ability to apply theoretical evidence to practice and make arguments and justifications for their clinical decisions that inform skill practices (Berndtsson, Dahlborg & Pennbrant 2020; Diery, Knogler & Seidel 2021). Younas and Quennell (2019) support nursing theory-guided practice as it assists to improve the quality of patient care, allowing students to articulate what they do for patients with clinical reasoning and judgement in applying evidence-based practice.

### The provision of holistic patient care

Holistic care is a comprehensive patient care model based on nurturing the wholeness and respect of patients’ uniqueness to physical, mental, emotional and environmental strengths and challenges, valuing their beliefs and health experiences (Rasweswe et al. 2021). When student nurses are engaged in a holistic approach to patient care, it enables them to incorporate a comprehensive and interprofessional sustainability that increases health outcomes. It further reduces medical errors and increases one’s purpose as a competent and caring professional. The provision of holistic patient care further leads to professional growth and builds the self-confidence of student nurses.

### The transition from novice to expert practitioners

The transition of student nurses’ proficiency from novice to expert develops over time from various forms of experiential learning and knowledge acquisition with an improved holistic understanding of the patient (Benner 1982; Ozdemir 2019; Gonzalez, Nielsen & Lasater 2021). Experiential learning and adequate exposure to various CLOs promote the students’ professional growth to make better clinical judgements and rational decisions, improving their practice over time to be confident in excellent patient care. Student nurses will practise competently, providing safe and quality patient care.

## The outcomes

### Autonomous professional practitioner

Student nurses begin exercising their professional autonomy as their knowledge and skills improve through clinical experiences and interactions within the interprofessional team approaches to quality care. To gain independence in professional practice, student nurses require adequate CLOs to develop and improve their clinical competencies and have the expertise and authority to make decisions following their professional knowledge base (Hara, Asakura & Asakura 2020; Labrague, McEnroe-Petitte & Tsaras 2019; Rouhi-Balasi et al. 2020). Autonomous healthcare professionals are also competent in critical thinking, with abilities to make discretionary decisions within their scope of practice and professional accountability for their actions and confidence (Oshodi et al. 2019).

### Confident professional practitioner

Student nurses gain professional confidence over time during their clinical experiential and interactive learning practices. The benefit of developing confident student nurses is to foster their belief in the ability to deliver safe and effective care while performing a complex role competently and skilfully within an interprofessional collaborative environment (Makarem et al. 2019; Owens & Keller 2018).

### Competent professional practitioner

The need to develop competent healthcare practitioners is vital to integrate the professional attributes that include knowledge, psychomotor skills, clinical judgement, values and beliefs required to perform in a variety of clinical settings and to fulfil their professional responsibility through practice (Allvin et al. 2020; Fukada 2018; SANC 2013). Having professional competency is crucial as it leads to quality improvement of patient care, which is the ethical and professional responsibility of healthcare professionals. Competent healthcare practitioners can integrate theory into practice and engage in interprofessional collaboration and cooperative problem-solving of complex health problems in dynamic healthcare settings (Saifan 2021).

### Critical thinking skills

Critical thinking skills are essential elements for student nurses, acquired during real-life experiences in a complex learning environment, to develop the knowledge and make critical decisions in solving the health problems of the patients in safe, efficient and skilful interventions (Carter, Creedy & Sidebotham 2018). Such skills serve as crucial guide to apply diagnostic and clinical reasoning skills to solve complex and challenging health problems (Diamond-Fox & Bone 2021). Student nurses with critical thinking skills can apply their acquired knowledge and experience to identify patient’s health problems in order to make logical and reasonable decisions to ascertain possible solutions to solve health complexities impacting patient’s outcomes. Poor critical thinking skills lead to student nurses’ poor interpretations of findings, inaccurate clinical judgement associated with incompetency and unsafe health practices (Antonova, Pletyago & Ostapenko 2020)

### Clinical reasoning skills

With adequate exposure to collaborative CLOs and problem-based activities, student nurses will learn and apply the acquired clinical knowledge, balance evidence and draw from their experiences to reach a definitive diagnosis of patients’ conditions (Wosinskic et al. 2018). The need of student nurses to develop sound clinical reasoning is to make the correct timeous diagnosis, apply critical thinking to determine interventions to reduce medical errors and ultimately improve the patient’s health outcomes (Barratt 2018; Kavanagh & Sharpnack 2021).

### Clinical judgement

Clinical judgement is one of the vital attributes of professional practice. It is related to critical thinking and clinical reasoning employed by healthcare professionals to conclude observations, analyse data and weigh evidence to make informed clinical decisions to improve patient care (Manetti 2018; Wosinskic et al. 2018). Engaging student nurses in complex real-life problem activities and research during their clinical learning helps them to apply their knowledge, develop critical thinking skills and practise evidence-based care that influences their independent clinical judgement.

Poor clinical judgement abilities result in more than half of adverse clinical events, delivery of poor and unsafe clinical practices and substandard patient care (Alfaro-LeFevre 2020). Hence, the need to enhance CLO for the student nurses to develop their good clinical judgement skills in applying their knowledge, experience, evidence and critical thinking skills in their daily practices.

### Critical decision-making skills

As the provision of care is becoming increasingly complex and challenging in the ever-changing health environment, the need to develop student nurses with critical decision-making skills during clinical learning and experiences is vital for their effectiveness in clinical practice and profession. Adequate exposure and engaging student nurses in complex real-life problem activities will enhance their ability to respond to changing circumstances, apply their knowledge and skills in different clinical situations to diagnose health problems and solve them accurately (Ahmady & Shahbazi 2020; Nibbelink & Brewer 2018).

### Problem-solving skills

Student nurses rely on CLOs to develop expertise and experiences in various situations to be equipped with problem-solving skills to solve complex health problems much more effectively and efficiently (Ahmady & Shahbazi 2020; Fatma, Sehrinaz & Tennur 2020). The varied and ever-changing healthcare environment requires healthcare professionals who are competent and able to identify real-life health problems and think creatively and collaboratively to integrate their knowledge to find possible health solutions to improve patients’ health.

## Conceptual map

**FIGURE 2:**
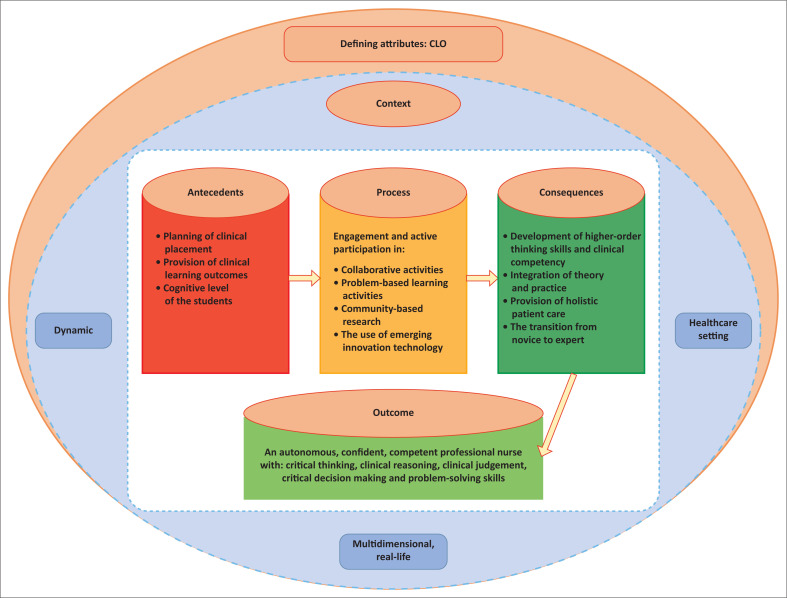
Conceptual map of clinical learning opportunity (CLO).

Conceptual mapping is a graphic presentation that signifies and depicts the relationships between concepts and ideas (Kunberger 2018). The lines and arrows in between explain the connections and relationships among the concepts. Figure 2 illustrates the conceptual mapping of concept analysis of clinical learning opportunity.

## Theoretical definition of clinical learning opportunity

The result of concept analysis was the identification of the defining attributes of clinical learning opportunity, which were used to develop and provide an operational definition with a clear theoretical base. The aim was to provide a clear understanding and consistency in the use of the concept within the study (Gray, Grove & Sutherland 2020; Walker & Avant 2019).

### Theoretical definition

The clinical learning opportunity is an occasion that occurs in dynamic, multidimensional, real-life healthcare settings that requires planning of clinical placement, provision of clinical learning outcomes and consideration of the cognitive level of students. There is engagement and active participation in collaborative, problem-based, community-based research activities and the use of emerging innovative technologies. It enhances the integration of theory and practice and develops HOTS and clinical competency to provide holistic patient care. It also supports the transition from novice to independent, confident practitioners with critical thinking, clinical reasoning and judgement, critical decision-making and problem-solving skills.

## Theoretical validity

Theoretical validity is the extent to which a concept is described and interpreted accurately. It further addresses the explicit theoretical construction that the researcher brings to the study (Hayashi, Abid & Hoppen 2019). The researcher consulted literature and multiple data sources (not limited to nursing) over an extended period to avoid a biased understanding of the true nature and meaning of the concept. Extensive literature was explored and critically analysed until data saturation. Similar and contrary patterns and phrases related to the concept were identified and clustered together to gain a better understanding of the concept.

Meaningful statements of clinical learning opportunity were objectively synthesised, transposed, redefined and classified within the context of the study. A theoretical and operational definition that consists of all the defining attributes was formulated to provide an accurate description of the concept. A model case was constructed to highlight the significance and practicality of the defining attributes within real-life clinical contexts. A contrary case was also formulated to avoid conflicting and contradictory understanding of the concept.

## Implications

Theoretical definition of clinical learning opportunity was developed to add to the existing knowledge in nursing education, practice and research. Without a well-constructed conceptual definition, the progression from conceptual understanding to a well-developed operational definition cannot take place. The article highlights the fundamental need for a concept analysis process that promotes a scientific approach in research to develop strategies to enhance positive clinical learning and professional development of student nurses within the healthcare learning environment and nursing education.

## Conclusion

The strategies to enhance the CLOs for student nurses in a public academic hospital will be developed, implemented and evaluated using the identified defining attributes and empirical referents of the concept. It is also recommended that the same defining attributes and empirical referents may be standardised and measured across nursing education by prospective researchers. The development of a conceptual definition could lead other researchers to develop and confirm an instrument to quantify the phenomenon and to compare data accurately and consistently across populations and over time. The results of concept analysis may further provide evidence to inform policymakers in the development of policies and guidelines for nursing education and clinical learning of student nurses.
